# Social Anxiety, Stress Type, and Conformity among Adolescents

**DOI:** 10.3389/fpsyg.2016.00760

**Published:** 2016-05-20

**Authors:** Peng Zhang, Yanhe Deng, Xue Yu, Xin Zhao, Xiangping Liu

**Affiliations:** ^1^School of Psychology, Beijing Normal University, BeijingChina; ^2^Behavior Rehabilitation Training Research Institution, School of Psychology, Northwest Normal University, LanzhouChina

**Keywords:** strong conformity, stressor type, social anxiety, adolescents, interaction

## Abstract

Social anxiety and stress type can influence strong conformity among adolescents; however, the interaction between them is not clear. In this study, 152 adolescents were recruited and assigned one of two conditions: an interaction and a judgment condition. In the interaction condition, adolescents with high social anxiety (HSA) were less likely to conform when completing a modified Asch task, compared to adolescents who had low social anxiety. In the judgment condition, adolescents with HSA were more likely to conform to the opinions from the unanimous majority. The results suggest that adolescents with HSA may show different styles of strong conformity with the change of stress type. We believe that socially anxious adolescents avoid potential social situations with weaker conformity, while avoiding negative evaluations from others with stronger conformity. These findings contribute to a better understanding of the social dysfunctions among adolescents with HSA and provide a new direction for clinical interventions.

## Introduction

Conformity refers that people keep their opinions or behaviors in accordance with the majority (Asch, 1956). According to formal evolutionary theory, conformity plays an important role in helping persons adapt to social lives (Boyd and Richerson, 1985; Rogers, 1988; Cialdini and Goldstein, 2004). Sometimes, conformity is naïve and people’s choices are unconsciously influenced (e.g., choosing one clothing style over another; Chartrand and Bargh, 1999). Other times, pre-established convictions influence behavior; however, we still tend to copy the performance of the unanimous majority (Henrich and Boyd, 1998; Henrich and Henrich, 2007). Researchers use the term *strong conformity* to distinguish it from naïve conformity (Haun and Tomasello, 2011).

Considerable research has indicated that adolescents show adaptable strong conformity to learning social skills effectively (Fusaro and Harris, 2008; Corriveau and Harris, 2009; Chan and Tardif, 2013; Chen et al., 2013). For example, Haun and Tomasello (2011) revealed that 4-year-old children expressed strong conformity to peer stressors. Similarly, Ma and Ganea (2010) found that children submitted to adults’ opinions when they were in conflict. Moreover, strong conformity can last consistently in children aged 3–17 years (Walker and Andrade, 1996).

However, recent research reported that social anxiety leads adolescents to exhibit non-adaptable conformity behaviors and other problems (Stewart et al., 2006; Ham et al., 2007; French et al., 2008; Lewis et al., 2008; Terlecki and Buckner, 2015). Social anxiety disorder is a mental disorder that consists of avoiding social situations and fearing negative evaluations (American Psychiatric Association [APA], 2013). Lewis et al. (2008) explored the relationship between social anxiety, alcohol consumption, and conformity motivation. Results indicated that highly socially anxious adolescents had stronger conformity motives toward using alcohol. Not surprisingly, this type of conformity comprises more negative consequences than benefits. Other studies have demonstrated that, these adolescents also expressed a tendency toward non-adaptable conformity when using alcohol (Stewart et al., 2006; Ham et al., 2007; Buckner and Shah, 2015; Terlecki and Buckner, 2015). In addition, social anxiety could exert influences on adolescents’ tendency to conform when recalling post-event information. Many studies have found that post-event information is affected by the unanimous majority, whereas adolescents with high social anxiety (HSA) showed a polarized orientation (Principe and Ceci, 2002; Cuc et al., 2006; Candel et al., 2007; French et al., 2008; Ost et al., 2008; Skagerberg and Wright, 2008). Wright et al. (2010) found that adolescents who avoided social situations were not easily affected by peers’ stress; however, adolescents who feared negative judgments were susceptible to peers. This polarization caused adolescents to display a lower correct rate when they recalled post-event information.

Above all, social anxiety can alter the strong conformity style among adolescents. However, existing research has shown that there is an inconsistent behavior pattern between social interaction stress (SIS) and social judgment stress (SJS) among children and adolescents with HSA (La Greca et al., 1988; Turner et al., 1989; Teachman and Allen, 2007). SIS refers to the possibility that individuals need to work together to accomplish something or to communicate effectively. SJS refers to an individual’s performance being observed, compared, and evaluated by others. Children show more strong conformity behaviors when they know that their own selection would be presented in public (Haun and Tomasello, 2011). Moreover, adolescents with HSA exhibited consistent avoidance when faced with social stressors, and thus were not susceptible to the unanimous majority. However, adolescents with HSA tend to copy the unanimous majority because they fear negative evaluations and attempt to avoid potential interpersonal conflicts (Wright et al., 2010). But there is no study to distinguish SIS from SJS. Accordingly, we hypothesize that adolescents will express a moderate level of strong conformity in both SJS and SIS conditions, and that an interaction between social anxiety and stressor will occur. Relative to adolescents with low social anxiety (LSA), adolescents with HSA will express less strong conformity under the SIS condition and more under the SJS condition.

To address the aforementioned problems, we enrolled adolescents aged between 10 and 16 years as participants. We chose this age range because these adolescents are in a critical stage of rapid development, are highly self-aware, and are a high-risk group for social anxiety (Morgan and Banerjee, 2006; Caouette and Guyer, 2014; Zhao et al., 2014). We utilized the Social Anxiety Scale for Children (SAS-C) to measure participants’ level of social anxiety. In addition, we evaluated their pre-established convictions. This test helped us ensure the final strategy was caused by the unanimous majority. Therefore, we determined that the participants’ conformity behaviors in the modified Asch task were examples of strong conformity. Then we conducted a modified Asch computer paradigm and asked them to make judgments of figures under the SIS and SJS conditions. We used three classmates as the unanimous majority because previous research has shown that three was sufficient to evoke conformity stress (Asch, 1956).

## Materials and Methods

### Participants

All participants were recruited from a primary school and a middle school (public and urban) in Gansu province, China. We distributed 182 copies of the SAS-C. One-hundred sixty-seven adolescents submitted valid questionnaires and their parents or legal guardians consented that they could take part in the next phase of the experiment. All participants were of Han nationality and were aged between 10 and 16 years (*M* = 13.01, *SD* = 1.49) with normal or corrected to normal vision, right-handed, and had no history of color blindness, neurological problems, or psychotherapy. Participants completed all procedures and they were rewarded with $5.

### Measures

#### SAS-C

The SAS-C was developed by La Greca et al. (1988). It is comprised of 10 items rated on a 3-point Likert-style scale (0 = Never, 1 = Sometimes, 2 = Always). Children’s anxiety was assessed on a score from 0 to 20, with higher scores indicating more severe social anxiety. The scale has two dimensions: a fear of negative evaluation (items = 1, 2, 5, 6, 8, and 10) and social avoidance (items = 3, 4, 7, and 9). The Chinese version of the SAS-C has sound reliability and validity, and it showed good internal consistency in this study (integral: α = 0.670; fear of negative evaluation: α = 0.646; social avoidance: α = 0.744).

#### The Modified Asch Paradigm

**FIGURE 1:**
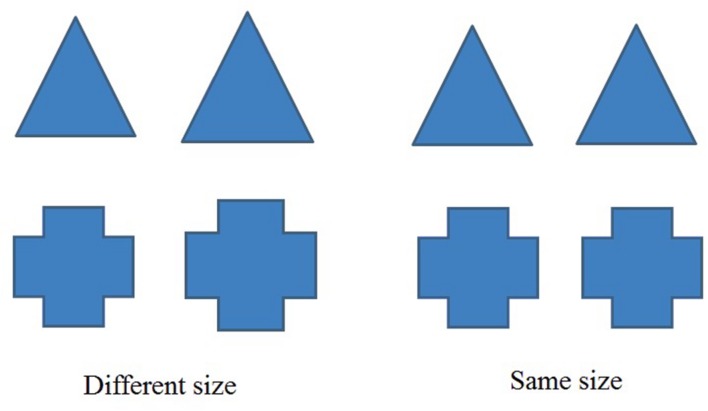
**Examples of pair-pictures**.

The instructions were presented first: “Please determine a pair of figures as same or different that will later be presented on the left part of your screen. The judgments of three classmates will be presented on the right part of your screen concurrently. You can consider their answers or ignore them; it is up to you. If you think the pair of figures is the same size, then press the ‘Y’ key on the keyboard; otherwise, press the ‘N’ key on the keyboard if you think they are different. Please press the ‘Q’ key to start when you are ready.” Afterward, a fixation point was presented centrally for 500 ms, and then a pair of figures was presented in the left part of the screen with the reminder presented in the right part of the screen until the participants responded. Then a blank appeared for 500 ms and the next trial began. These figures consisted of 12 pairs including six same-size pairs and six different-size pairs (see **Figure 1**). Each group of figures repeated twice. The judgments of the unanimous majority were the same when presented the different-size pairs and were the different when presented the same-size pairs. Each participant was required to complete 24 (12 pairs × 2 repetitions) trials in one session that lasted approximately 15 min. This procedure was processed by E-prime 2.0 (Psychology Software Tools Inc., Pittsburgh, PA, USA). The entire task was presented on a 19-inch display (PC, refresh rate = 70-Hz) with 1440 × 900 resolution with a black background, white instructions and reminders, and green figures. Participants sat approximately 60 cm from the monitor at a 3°angle.

### Procedure

The Beijing Normal University Ethics Committee approved all stages of this study.

All participants and their parents provided written informed consent. It took participants 3 days to finish the entire procedure. On the 1st day, participants completed the questionnaires in a quiet and spacious classroom with the help of research assistants. The questionnaires were pencil-and-paper tests and lasted approximately 5 min. Participants’ pre-established convictions were evaluated after they completed the questionnaire. Twelve figure-pairs (these were the same as those utilized in the modified Asch task, see **Figure 1**) were presented with a randomized sequence on the computer. Each participant was asked to judge whether the pair of figures was the same size. On the 2nd and 3rd day, all participants completed a session of a modified Asch task. To balance for order effects, we generated random numbers to assign participants into group A or group B. Group A comprised 82 participants and Group B comprised 83 participants. Group A completed the modified Asch task under the SIS condition on the 2nd day, and then completed it under the SJS condition on the 3rd day, whereas Group B did the opposite. For the SIS condition, participants were told, “You will complete a task that requires you to judge the size of figures with your classmates. Please try to make the correct selection. You will see the responses made by three other classmates; however, no one can see your selection.” For the SJS condition, participants were told, “You will now complete a task that involves judging the size of figures independently. Please make the correct selection. You will see the selections made by three other classmates, and they will see yours.”

## Results

### Data Reduction

Data from 15 participants were excluded due to the following reasons: failure to understand the modified Asch task (*n* = 2), physical problems or a lack of time (*n* = 6), and error rate more than two standard deviations above the mean (*n* = 7). Therefore, the data of 152 participants (females: *n* = 73 *M_age_* = 13.01, *SD* = 1.66; males: *n* = 79 *M_age_* = 13.03, *SD* = 1.33) were included in data analysis. Data were processed using SPSS 19.0 (IBM Corporation, Armonk, NY, USA).

### Correlations between Social Anxiety and Conformity

The results of a pre-established convictions test showed that all participants had a 100% accuracy rate in the judgment of 12 pairs of figures; therefore, if participants made a wrong response in the modified Asch task it implied strong conformity. Results indicated that adolescents showed strong conformity to the unanimous majority under both the SIS and SJS conditions. There was a significantly negative correlation between social anxiety and strong conformity under the SIS condition and a significantly positive correlation under the SJS condition (see **Table 1**). It revealed that adolescents expressed less strong conformity under the SIS condition, and that more under the SJS condition with the increase of social anxiety. This is consistent with our hypothesis.

**Table 1:** Correlations between social anxiety and conformity (*n* = 152).

	*M*	*SD*	1	2	3	4
(1) Ages	13.01	1.49				
(2) Education years	7.04	1.16	0.681ˆ***			
(3) Social anxiety	7.14	3.44	-0.155	-0.183ˆ*		
(4) Social interaction stress (SIS)	0.486	0.105	0.067	0.012	-0.248ˆ**	
(5) Social judgment stress (SJS)	0.480	0.111	0.081	0.086	0.211ˆ**	0.122

*p < 0.05, **p < 0.01, ***p < 0.001.

### Group Analysis

To further explore the interaction of social anxiety and stress type on strong conformity, we selected the participants who obtained a social anxiety score in the top 27% of the HSA group (*n* = 41), and in the bottom 27% of the LSA group (*n* = 41; Gibson and Dembo, 1984). A *t*-test showed that there was a significant difference of participants’ error rate in the modified Asch task between conditions. This indicated that our stress-type manipulation was effective (LSA: *t* = 2.024, *p* = 0.046; HSA: *t* = -3.190, *p* = 0.002). Upon examination, there were no significant differences in age and education. Social anxiety and error rate in SIS and SJS showed significant differences in both groups (see **Table 2**). The χ^2^ test results showed that there were no significant differences for the number of males and females in either group [χ^2^(82) = 0.195, *p* = 0.825].

**Table 2:** Group differences in statistics (*n* = 82).

	Low social anxiety (LSA; Female = 20)		High social anxiety (HSA; Female = 22)		*t*	*p*
	*M*	*SD*	*M*	*SD*
Ages	13.32	1.33	13.63	1.50	0.821	0.117
Education years	7.00	1.57	6.84	1.56	1.225	0.222
Social anxiety	2.83	1.88	11.34	1.30	-23.850	0.000
SJS	0.461	0.102	0.513	0.099	3.035	0.003
SIS	0.498	0.091	0.441	0.104	-2.340	0.022

**FIGURE 2:**
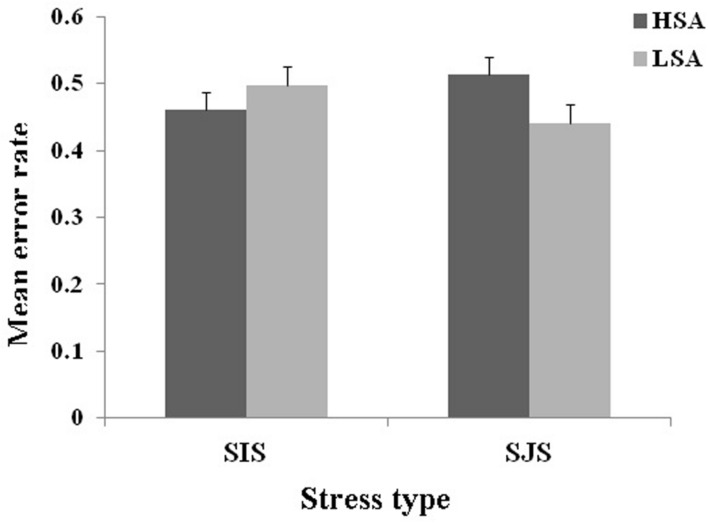
**Mean error rate on the modified Asch task under the SIS or SJS conditions, for HSA and LSA adolescents**.

Furthermore, we used a two-way ANOVA to compare the mean differences between error rates that had been split across our two independent variables (Group and Stress Type). Neither the main effect of Group [*F*(1,163) < 1] nor Stress Type [*F*(1,163) = 1.372, *p* = 0.243, $\eta_{p}^{2}$ = 0.009] was significant. However, the Group × Stress Type interaction was significant [*F*(3,163) = 14.052, *p* < 0.001, $\eta_{p}^{2}$ = 0.081] (see **Figure 2**). Further simple effects of Group showed a higher error rate in HSA than LSA under the SJS condition [*F*(1,160) = 6.353, *p* = 0.038, $\eta_{p}^{2}$ = 0.013], and a lower error rate in HSA than LSA under the SIS condition [*F*(1,160) = 7.733, *p* = 0.046, $\eta_{p}^{2}$ = 0.006]. It indicated that adolescents with HSA showed a higher probability of strong conformity behavior than LSA in the SJS condition (*M*_HSA_ = 0.513, *M*_LSA_ = 0.461, *p* = 0.022), and a lower probability of strong conformity behavior than LSA under the SIS condition (*M*_HSA_ = 0.441, *M*_LSA_ = 0.498, *p* = 0.003). This is consistent with our hypothesis. We found a significant sex difference for strong conformity; however, our results were not affected by these sex differences^1^.

## Discussion

This study utilized a modified Asch paradigm to measure the effects of social anxiety and stress type on strong conformity in adolescents aged between 10 and 16 years. As predicted, when controlling for variables such as age and education, we found that adolescents showed strong conformity to the unanimous majority under both the SIS and SJS conditions. There was a significant interaction of social anxiety and stress type effects on strong conformity in adolescents. Specifically, adolescents with HSA decreased their strong conformity during the SIS condition, whereas they increased it during the SJS condition compared to adolescents with LSA.

Regardless of condition, all adolescents expressed strong conformity to the unanimous majority, which was consistent with existing literature (Fusaro and Harris, 2008; Corriveau and Harris, 2009; Hanayama and Mori, 2011; Haun and Tomasello, 2011; Chan and Tardif, 2013; Chen et al., 2013). Researchers feel that strong conformity is an important, adaptable strategy among individuals’ developing process (Morgan et al., 2015). Perhaps natural selection makes us respect others’ ideas and consider them to aid our survival rate. Those keeping accordance with others are more likely to obtain acceptance and protection from the group; therefore, they are more likely to survive. In particular, with the increasing importance of group social connections, a strong conformity pattern has a more realistic meaning.

Second, we found a significant interaction of social anxiety and stress type effects on strong conformity in adolescents. Compared to adolescents with LSA, adolescents with HSA showed less strong conformity during the SIS condition and more strong conformity during the SJS condition. Previous research had confirmed that socially anxious people showed different behaviors during situation changes (La Greca et al., 1988; Turner et al., 1989; Teachman and Allen, 2007). Our study reinforced the effects of different situations on the behavior of socially anxious individuals. During the SIS condition, adolescents with HSA showed more strong conformity and a tendency to avoid peers unconsciously, although the avoiding behavior was not observed directly in this study. This implies that social avoidance among adolescents with HSA is not limited to just observable behavior, but also to internalized cognitions and to social decision-making. This avoidance style may alleviate anxiety; however, it reduces the opportunity to interact with others, which makes them at a disadvantage in social interactions (Stein and Stein, 2008). During the SJS condition, adolescents with HSA showed less strong conformity to the unanimous majority. On one hand, this may help them obtain the group’s acceptance and avoid potential interpersonal conflicts. On the other hand, it exerts negative effects on an individuals’ self-esteem. Previous literature has validated the importance of low self-esteem on the induction and persistence of social anxiety (Leary, 1990; de Jong, 2002; Glashouwer et al., 2013; Sowislo and Orth, 2013; van Tuijl et al., 2014). Indeed, excessive strong conformity leads to lower self-esteem, which further leads to higher levels of social anxiety. This adverse cycle makes adolescents with HSA more likely to develop dysfunctional strong conformity.

This study has some limitations. First, the experimental situations including SIS and SJS require validation. Although the data indicated that our stress-type manipulation was effective, more evidence is necessary to determine the contribution of this method. Second, the induction and persistence of social anxiety was affected by considerable variables such as genetics, neural damage, social support, depression, and self-esteem (Stein and Stein, 2008). Future research should include more variables to improve the credibility of the current conclusions. Finally, the neural mechanism underlying this behavioral pattern has not yet been revealed. Further studies should use fMRI, ERPs, etc., to explore these mechanisms.

Although this study has limitations, it also provided a novel way of understanding the non-adaptive strong conformity of adolescents with social anxiety. On the first, We emphasized the importance of distinguishing between the two types of stressors. Adolescents with HSA showed less conformity under the SIS condition but more conformity under the SJS condition. Different types of stress represent different source of social anxiety, leading to different styles of strong conformity. This results help us understand how adolescents with HSA show different conform style with the changes of stress. Secondly, in terms of clinical intervention, we should aware the influence of social anxiety on strong conformity, which may has different processes. Finally, cognitive training aiming to promote self-esteem may help adolescents with HSA evaluate which is more effective, holding their own opinion or conforming to the majority.

## Author Contributions

PZ and XL designed research and analyzed data; XZ, YD, and XY wrote the paper. All authors contributed and have approved the final manuscript.

## Conflict of Interest Statement

The authors declare that the research was conducted in the absence of any commercial or financial relationships that could be construed as a potential conflict of interest.

## Footnotes

1 Females (*M* = 0.499, *SD* = 0.093) showed higher conformity tendency than males did (*M* = 0.469, *SD* = 0.095, *p* = 0.021). We separated females and males for data analysis. For males, adolescents with HSA showed stronger conformity behavior than LSA in the SJS condition (*M*_HSA_ = 0.485, *M*_LSA_ = 0.440, *p* = 0.047), and weaker conformity behavior than LSA in the SIS condition (*M*_HSA_ = 0.452, *M*_LSA_ = 0.496, *p* = 0.068). For females, adolescents with HSA showed stronger conformity behavior than LSA in the SJS condition (*M*_HSA_ = 0.541, *M*_LSA_ = 0.482, *p* = 0.024), and weaker strong conformity behavior than LSA in the SIS condition (*M*_HSA_ = 0.430, *M*_LSA_ = 0.501, *p* = 0.006). Above all, we ensured that females and males showed the same style of strong conformity in both conditions. This indicated that the results were stable and reliable.
